# Antiproliferative and apoptotic effects of proteins from black seeds (*Nigella sativa*) on human breast MCF-7 cancer cell line

**DOI:** 10.1186/s12906-019-2804-1

**Published:** 2020-01-13

**Authors:** Yamna Khurshid, Basir Syed, Shabana U. Simjee, Obaid Beg, Aftab Ahmed

**Affiliations:** 1 Biomedical and Pharmaceutical Sciences, Chapman University School of Pharmacy, 9401 Jeronimo Road, Irvine, CA 92618 USA; 2 Dr. Panjwani Center for Molecular Medicine and Drug Research, International Center for Chemical and Biological Sciences, University of Karachi, Karachi, 75270 Pakistan

**Keywords:** *Nigella sativa* seed, Protein purification, Anticancer activity, Apoptosis, LC-MS/MS

## Abstract

**Background:**

*Nigella sativa* (NS), a member of family Ranunculaceae is commonly known as black seed or *kalonji*. It has been well studied for its therapeutic role in various diseases, particularly cancer. Literature is full of bioactive compounds from NS seed. However, fewer studies have been reported on the pharmacological activity of proteins. The current study was designed to evaluate the anticancer property of NS seed proteins on the MCF-7 cell line.

**Methods:**

NS seed extract was prepared in phosphate-buffered saline (PBS), and proteins were precipitated using 80% ammonium sulfate. The crude seed proteins were partially purified using gel filtration chromatography, and peaks were resolved by SDS-PAGE. MTT assay was used to screen the crude proteins and peaks for their cytotoxic effects on MCF-7 cell line. Active Peaks (P1 and P4) were further studied for their role in modulating the expression of genes associated with apoptosis by real-time reverse transcription PCR. For protein identification, proteins were digested, separated, and analyzed with LC-MS/MS. Data analysis was performed using online Mascot, ExPASy ProtParam, and UniProt Knowledgebase (UniProtKB) gene ontology (GO) bioinformatics tools.

**Results:**

Gel filtration chromatography separated seed proteins into seven peaks, and SDS-PAGE profile revealed the presence of multiple protein bands. Among all test samples, P1 and P4 depicted potent dose-dependent inhibitory effect on MCF-7 cells exhibiting IC_50_ values of 14.25 ± 0.84 and 8.05 ± 0.22 μg/ml, respectively. Gene expression analysis demonstrated apoptosis as a possible cell killing mechanism. A total of 11 and 24 proteins were identified in P1 and P4, respectively. The majority of the proteins identified are located in the cytosol, associate with biological metabolic processes, and their molecular functions are binding and catalysis. Hydropathicity values were mostly in the hydrophilic range.

**Conclusion:**

Our findings suggest NS seed proteins as a potential therapeutic agent for cancer. To our knowledge, it is the first study to report the anticancer property of NS seed proteins.

## Background

Breast cancer is the leading cause of cancer-related death in women accounting for 2.1 million new cases and 627,000 deaths globally in 2018 according to the last release of GLOBOCAN data [1]. Considering the fact of increasing burden and downside of current therapies available for breast cancer, search for new effective drugs is important. One most prominent hallmark in all malignant cells is evasion from apoptosis and is triggered either by the intrinsic mitochondrial pathway or extrinsic death receptor-mediated pathway [2]. Irrespective of the pathway involved in apoptosis, the execution is mediated by a cascade of cysteine-aspartic proteases named as caspases that are further categorized into initiator caspases (caspase 2, 8, 9 and 10) and executioner caspases (caspase 3, 6 and 7). Therefore, inducing apoptosis is a pivotal approach in fighting against tumor [3].

Historically, compounds derived from medicinal plants serve as a promising source of pharmaceuticals for numerous ailments. According to the World Health Organization (WHO), more than 80% of the population around the globe depend entirely on medicinal plants and herbal remedies for their treatment [4]. Plant-derived compounds account for more than 60% of the approved anticancer drugs [5, 6], including taxol, vincristine, vinblastine, and camptothecin derivatives [7]. Despite years of research and many treatment options available toxicity, off-targets, drug resistance, and limited bioavailability remain a challenge on the way to complete cure. With the advancement in proteomics, protein-based drugs emerged out as a potential anticancer agent [8]. These drugs offer many advantages over small molecules as they are highly potent and selective therefore, less toxic [9]. Medicinal plants are a rich source of biologically active proteins and peptides particularly those belonging to Solanaceae, Ranunculaceae, Brassicaceae, Asteraceae, Fabaceae, and Cucurbitaceae families [10]. Evaluation of protein and peptides from these families for their anticancer potential could provide a piece of preliminary information for the selection of plants with potent antitumor activity for future studies.

*Nigella sativa* (NS), a medicinal herb frequently known as black seed or *Kalonji,* is a member of a Ranunculaceae family, well known for its pharmacological action against various human diseases [11]. Its cultivation is native to Southwest Asia, Southern Europe, and North Africa [12]. The seeds of NS reported to contain fats (28.5%), proteins (26.7%), carbohydrates (24.9%), crude fibers (8.4%) and ash content (4.8%) [13, 14]. To date, more than 100 bioactive compounds, including thymoquinone, alkaloids (nigellidine, nigellicine), phenol (carvacol), and saponin (alpha-hederin) have been reported from NS [15]. These compounds have been shown to possess a wide range of activities, including anticancer, antimicrobial, anti-diabetic, analgesic, immunomodulatory, diuretic, antihypertensive, hepatoprotective, spasmolytic, gastroprotective, renal protective, a bronchodilator, and antioxidant properties [16]. However, limited literature is available on the pharmacological activity of NS seed proteins. Recently, two novel thionins named NsW1 and NsW2 reported potent against *Staphylococcus aureus, Bacillus subtilus* and *Candida albicans* [17]. Previously, isolated defensin peptides, Ns-D1, and Ns-D2 have also shown a strong inhibitory response against various strains of phytopathogenic fungi [18]. To the best of our knowledge, proteins from NS seeds have not been studied for their anticancer potential yet. Hence, the present study was designed to demonstrate the effect of NS seed protein extract and partially purified proteins on human MCF-7 breast cancer cell line. Their effect on the expression of pro- and anti-apoptotic genes is also investigated to determine tumor cell killing mechanisms. Furthermore, active proteins were identified using high-resolution mass spectrometer and classified based on their cellular compartment, biological process, and molecular functions.

## Method

### Plant material

NS seeds were bought from the local grocery store of Karachi, Pakistan. It was, authenticated based on morphology by Dr. Muneeba Khan, Taxonomist at Center for Plant Conservation, University of Karachi Herbarium, and Botanical Garden. Voucher specimen has been deposited, but no deposition number available. As per institutional policy, seeds are not accepted as a voucher specimen for submission; therefore, deposition number is issued for whole plants only. Debris and other particles were removed by handpicking, and seeds were grounded into a fine powder using a food processor.

### Protein extraction

The proteins were extracted using a previously described method with minor modification [19]. In brief, 200 g of seed powder was dissolved in phosphate-buffered saline (PBS) 1X, (1:10 w/v) pH 7.2, for overnight at 4 °C with continuous stirring. Following this, seed extract was centrifuged for 30 min at 4 °C and 10,000 rpm, the supernatant was separated, and 80% ammonium sulfate was added to the supernatant at 4 °C. The next day, the protein precipitates were obtained by centrifugation at similar conditions. The pellet was dissolved in PBS, dialyzed in deionized water, and lyophilized.

### Gel filtration chromatography

The crude seed protein extract was fractionated on ÄKTA start (GE Healthcare) fast protein liquid chromatography (FPLC) system equipped with a 280 nm detector and automated fraction collector. A 300 mg of crude seed protein sample was loaded onto Hiload™ 16/60 Superdex™ 200 pg gel filtration column (GE Healthcare) pre-equilibrated with PBS. All fractions were collected at a flow rate of 1 ml/min (5 ml in each tube), and absorbance was recorded at 280 nm.

### Protein estimation by Bradford assay

The crude seed proteins and gel filtration peaks were quantified using commercially available Coomassie Bradford assay kit (Thermo Fisher Scientific, USA) following the provided protocol. Briefly, 5 μl of standard bovine serum albumin and protein sample were added to 96-well plate, and 250 μl of Bradford reagent was added and mixed gently. All the samples were analyzed in triplicates. After 10 min of incubation, absorbance was measured at 595 nm wavelength using a microplate photometer.

### Sodium dodecyl sulfate-polyacrylamide gel electrophoresis (SDS-PAGE)

The crude proteins of NS seed and gel filtration chromatography peaks were resolved on 4–20% Tris/glycine precast Mini- PROTEAN TGX gels (Bio-Rad) using Mini-PROTEAN Tetra Cell assembly (Bio-Rad). A 15 μl of 2X Laemmli sample buffer (Bio-Rad) containing 2-mercaptoethanol was mixed with 15 μl (20 μg) of sample, heated at 95 °C, and run at a constant volt of 200 V for 45 min. To visualize protein bands, the gels were stained with Coomassie Blue R-250 [20].

### Cell culture

The human breast cancer cell line MCF-7 (ATCC HTB-22™) used in this study was obtained from Dr. Panjwani Center for Molecular Medicine and Drug Research (PCMD) Biobank. Cells were maintained in DMEM medium supplemented with fetal bovine serum (FBS) 10% (v/v) and penicillin-streptomycin 1% (v/v) in a humidified incubator at 37 °C containing 5% CO_2_.

### MTT assay for cell viability

MTT [3-(4, 5-dimethylthiazol-2-yl)-2, 5-diphenyl tetrazolium bromide] assay was utilized to measure the anti-proliferative potential of NS seed proteins [21]. Briefly, 3 × 10^4^ cells /ml were cultured in 96- wells plates in 200 μl of volume and incubated at 37 °C in 5% CO_2_ for 24 h. The next day, the medium was removed, and for screening, cells were treated with different concentrations (25, 50, 100, 200, and 400 μg/ml) of NS seed crude protein and gel filtration chromatography peaks for 48 h. As only peak 1 (P1) and peak 4 (P4) exhibited cytotoxic response against MCF-7 therefore, they are further selected for finding IC_50_ and treated at a dose of (10, 20, 40, and 60 μg/ml) for P1 and (5, 10, 20, and 40 μg/ml) for P4.In control cells, only medium was added, and doxorubicin was used as a standard drug. After 48 h, MTT dye (0.5 mg/ml) prepared in 200 μl medium was added to the individual well and incubated at 37 °C in 5% CO_2_ for 4 H*. medium* containing dye was removed after incubation, and purple formazan crystals were solubilized in 100 μl of DMSO. Absorbance was recorded at 550 nm wavelength using a Multiskan FC Microplate Photometer (Thermo Fisher Scientific, USA). The percentage of cell inhibition was calculated using the formula:

$$ \mathrm{Percent}\ \mathrm{Inhibition}=\frac{\left(\mathrm{O}.\mathrm{D}.\mathrm{of}\kern0.17em \mathrm{untreated}\kern0.17em \mathrm{cells}-\mathrm{O}.\mathrm{D}.\mathrm{of}\kern0.17em \mathrm{treated}\kern0.17em \mathrm{cells}\right)\;\mathrm{x}\;100}{\mathrm{O}.\mathrm{D}.\mathrm{of}\kern0.17em \mathrm{untreated}\kern0.17em \mathrm{cells}.} $$

RNA isolation and cDNA synthesis

MCF-7 1 × 10^6^ cells/well were seeded in a 6-well plate and placed in 37 °C incubator having 5% CO_2_. After 24 h, MCF-7 cells were treated with P1 (10 and 14 μg/ml) and P4 (5 and 8 μg/ml). The total RNA was isolated by using TRIzol reagent (Invitrogen) following the manufacturer’s instruction. The isolated RNA was run on 1.2% agarose gel electrophoresis for 45 min at 80 V to determine the integrity and quantified using NanoDrop 2000 Thermo Scientific spectrophotometer. Samples with high yield, good integrity, and purity ratio 260/280 of ~ 2 were selected for cDNA synthesis. cDNA was synthesized using the RevertAid First Strand cDNA synthesis kit (Thermo Scientific, USA) in Eppendorf® Mastercycler® Pro thermal cycler (USA) according to manufacturer’s instructions.

### Real time PCR for gene expression analysis

qPCR was performed in Stratagene Mx3000P (Agilent Technologies, USA) instrument by using Maxima SYBR Green/ROX qPCR master mix kit (cat# K0221; Thermo Scientific) as per manufacturer’s protocol. After amplification, melting curve analysis was performed by following the method; 95 °C for 1 min, 55 °C and 95 °C for 30 s each. GAPDH was used as a reference gene for the normalization of data. Fold change in gene expression was calculated with the CT values obtained by ∆∆ct method.

### In-solution digestion

The lyophilized proteins, 1 mg of P1 and P4, were solubilized separately in 100 μl of urea 6 M, and reducing agent 5 μl DTT (200 mM) was added. The samples were vortexed and incubated at room temperature for 1 h. An alkylating agent, 20 μl iodoacetamide (200 mM) was added, and the mixture was incubated at room temperature in the dark for 1 h. The reaction was stopped by adding 20 μl of DTT, and urea concentration was reduced by diluting it with 775 μl of MilliQ water. Digestion was carried out overnight (< 16 h) by adding TPCK treated trypsin in a ratio of 1:30 (Trypsin: Protein). The next day, digestion was stopped by adding 10 μl of acetic acid [22].

### Liquid chromatography and ultra-high-resolution mass spectrometry

The tryptic peptides were separated on BioZen 2.6 μm Peptide XB-C18 (pore size 100 Å, dimension 150 × 2.1 mm) column (Phenomenex) and analyzed using Impact II™ UHR-QqTOF (Ultra-High Resolution Qq-Time-Of-Flight) mass spectrometer (Bruker). The mobile phases used were 0.1% formic acid in water (solvent A) and 0.1% formic acid in acetonitrile (solvent B). The flow rate was adjusted to 0.3 ml/min with following 85 min gradient: from 0 to 5 min 1% B, 5–60 min 50% B, 61–65 min 50% B, 65–70 min 95% B, 71–78 min 95% B, 79–85 min 1% B.

### MS data analysis

Mascot generic files (mgf) generated from raw MS/MS files were used to search against the SwissProt-taxonomy Viridiplantae (39,582 sequences) database by using an in-house licensed Mascot search engine. Mascot search parameters were set as Carbamidomethylation of cysteine as a fixed modification, oxidation of methionine as variable modification, peptide mass tolerance allowed was ±1.2 Da, fragment mass tolerance allowed was ±0.6 Da and trypsin as an enzyme with a maximum of one mixed cleavage permitted. The false discovery rate (FDR) was set as 1%. Proteins identified with more than two matched peptides and greater than 99% probability were accepted.

### Gene ontology annotation and hydropathy profile

The gene ontology annotation of the identified proteins was obtained from the UniProt Knowledgebase (UniProtKB) gene ontology (GO) project (https://www.uniprot.org/uniprot/). The proteins were categorized based on their molecular functions, biological process, and cellular localization. To calculate the grand average of hydropathy (GRAVY) value, the FASTA sequence of the identified proteins was obtained from the UniProt database, and the GRAVY values were computed using online ExPASy tool ProtParam (https://web.expasy.org/protparam/).

### Statistical analysis

SPSS version 20.0 software was used to analyze the data. One-way ANOVA (analysis of variance) and Dunnett’s post-hoc test was used for comparing the treatment groups with control. Results with *P* values *P* < 0.05 were considered statistically significant whereas ***P < 0.001, **P < 0.01 and *P < 0.05.

## Results

### Partial purification of *Nigella sativa* seed proteins

**Fig. 1:**
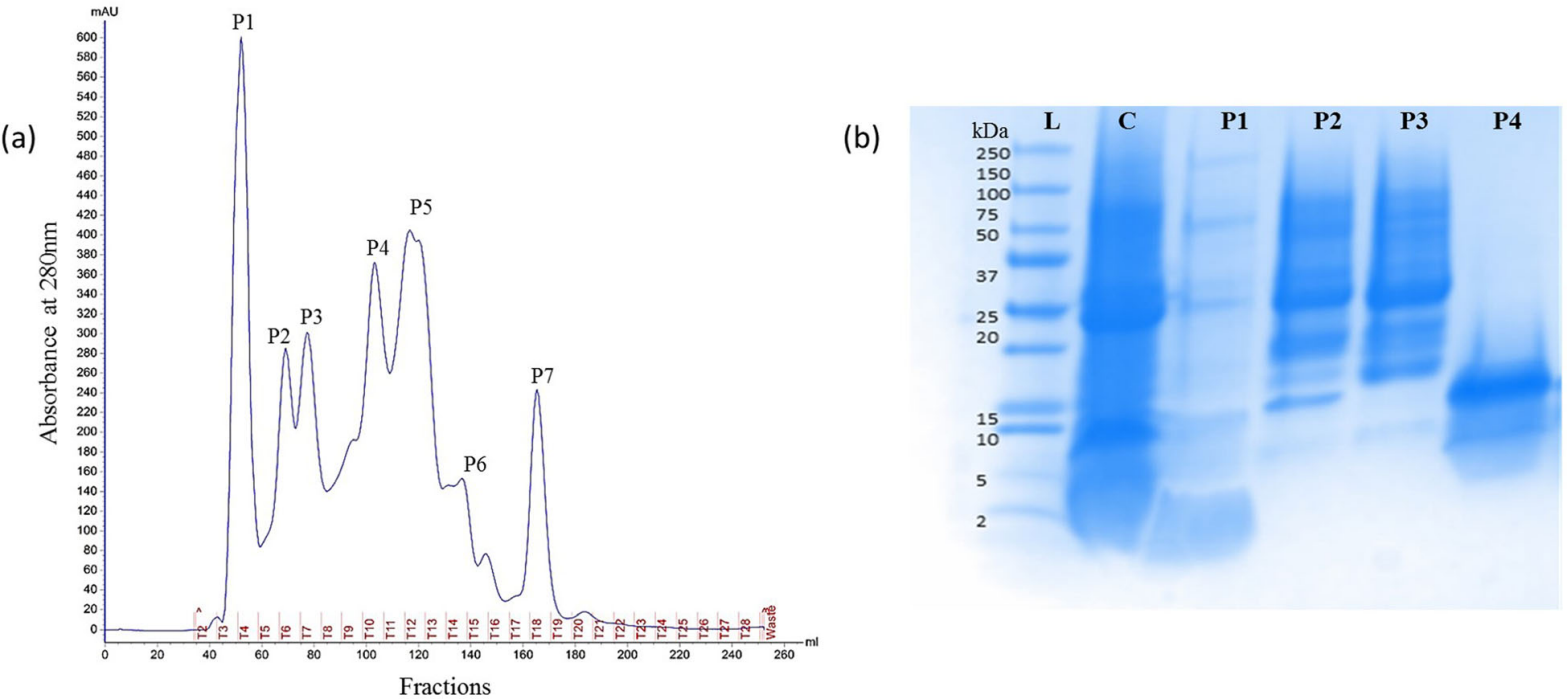
(**a**) Elution profile of gel filtration chromatography of protein extract *Nigella sativa* seeds. The column HiLoad 16/600 Superdex 200 pg pre-equilibrated with 1X PBS buffer. The flow rate was 1 ml/min, and absorbance monitored at 280 nm. (**b**) The electrophoretic profile on 4–20% Tris/glycine SDS PAGE gel. The samples analyzed crude seed proteins (C), gel filtration chromatography peaks (P1, P2, P3, and P4) and standard molecular weight ladder (L)

The crude NS seed proteins were successfully precipitated from the extract prepared in PBS using 80% ammonium sulfate. This step was employed to get rid of small molecules, including polysaccharides, nucleic acids, polyphenols, and pigments from the extract. Whereas, ammonium sulfate has no harmful effects on protein function and structure [23]. To evaluate the cytotoxic potential of the crude seed, precipitated proteins were partially purified on gel filtration chromatography column Hiload™ 16/60 Superdex™ 200 pg using ÄKTA start FPLC system. The proteins were eluted using an elution buffer PBS at 1 ml/min of flow rate, and absorbance was measured at 280 nm. The eluted fractions were collected 5 ml volume in each tube. Figure 1a shows the separation of the crude sample into seven peaks. All the peaks were collected and resolved on the gel. The Tris/glycine SDS-PAGE analysis of crude protein revealed the presence of several protein bands within a range of ~ 2–150 kDa (Fig. 1b). The SDS-PAGE profile clearly showed protein bands in a molecular weight range of ~ 5–150 kDa in peak 1 (P1), ~ 15–100 kDa in both peak 2 (P2) & peak 3 (P3) and ~ 5–15 kDa in peak 4 (P4) (Fig. 1b). Whereas peaks (P5, P6, and P7) did not show any protein band.

### Cytotoxic effect of *Nigella sativa* on human cancer cell line

**Fig. 2:**
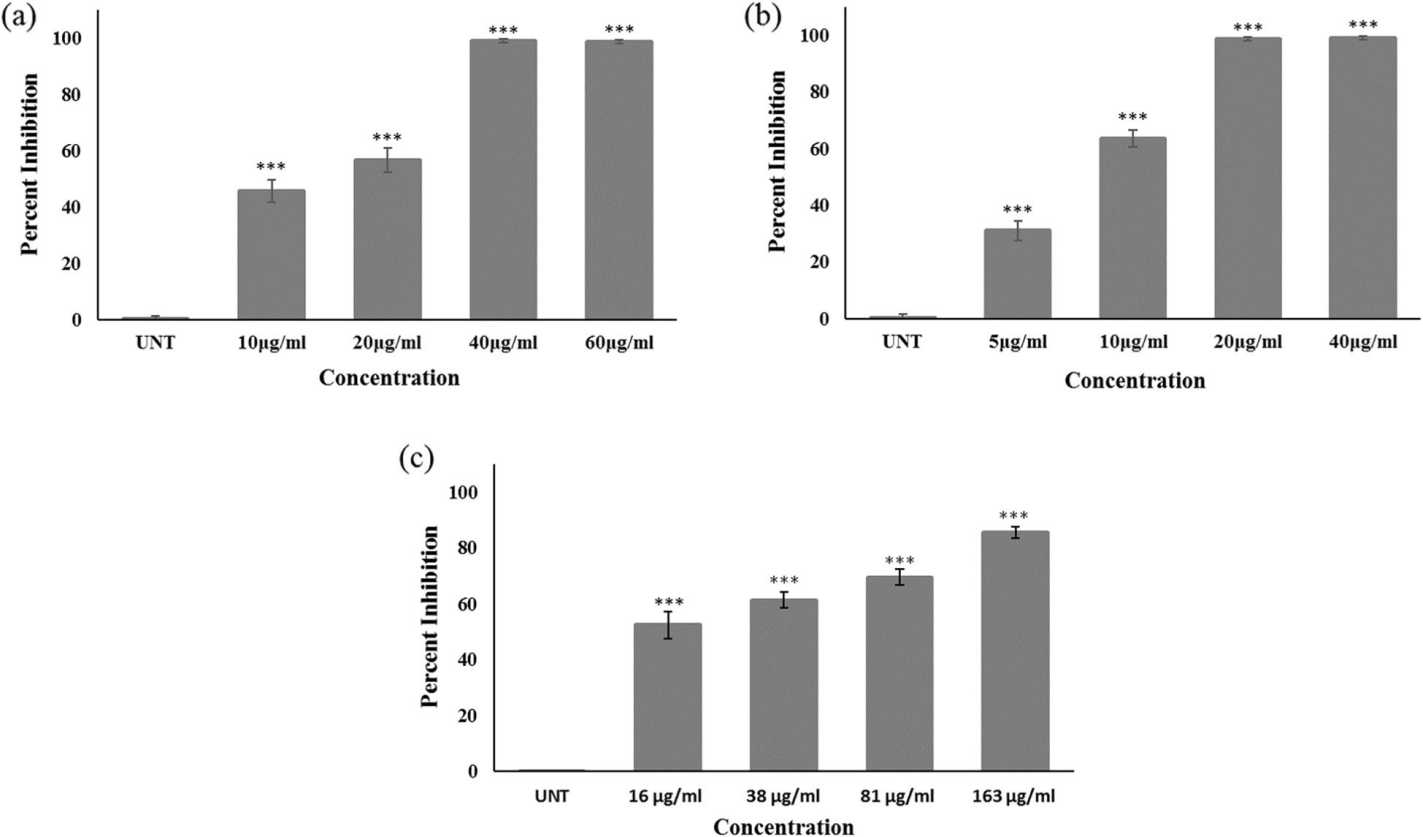
The dose-dependent cytotoxic effect of (**a**) P1 (**b**) P4 and (**c**) doxorubicin on MCF-7 cell line. The IC_50_ values calculated were 14.25 ± 0.84 for P1, 8.05 ± 0.22 for P4 and 16 ± 0.01 μg/ml for doxorubicin. Data are expressed as a mean of triplicates for each dose ± S.D. from three independent experiments. UNT labeled bar represents untreated control cells. The statistically significant values are labeled as *P < 0.05, **P < 0.01 and ***P < 0.001 as compared to the control

**Fig. 3:**
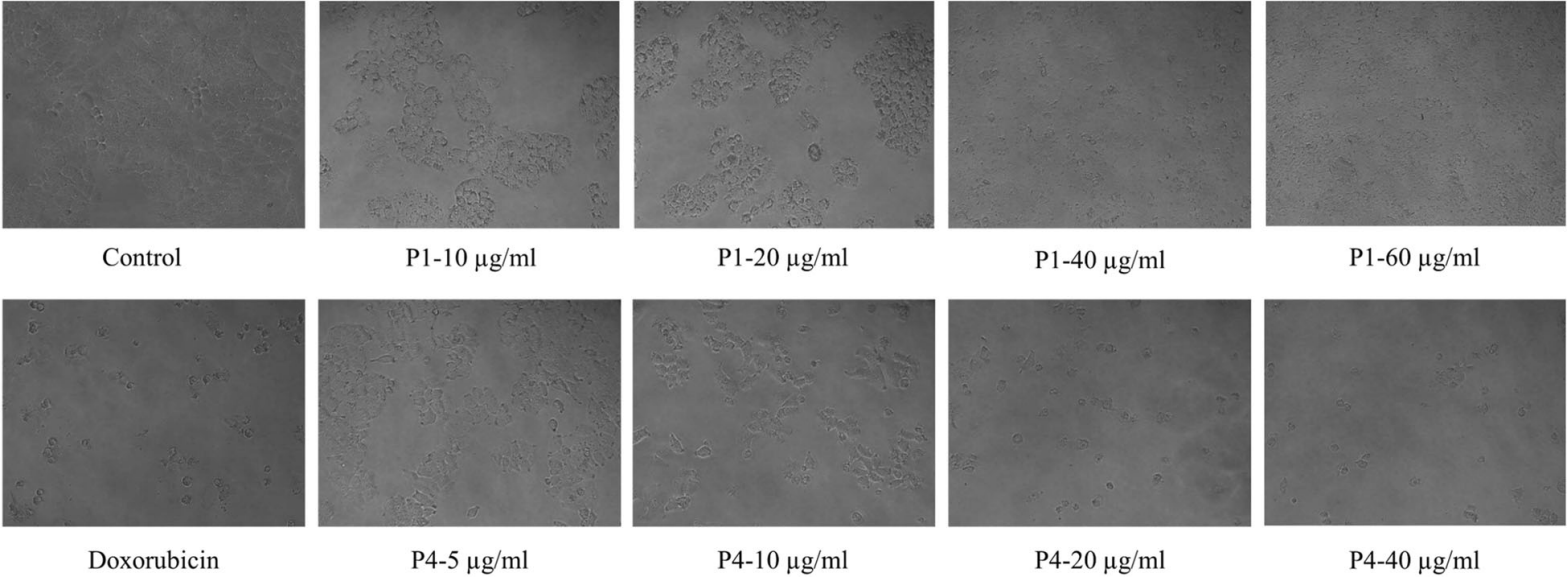
Phase contrast microscopy images of MCF-7 cells after treatment with P1 and P4

Prior to cytotoxic analysis, all the samples were desalted by ultrafiltration (3 kDa MWCO) and quantified by Bradford assay. All the seven peaks and crude seed proteins were screened at different doses (25, 50, 100, 200, and 400 μg/ml) for their effect on the viability of MCF-7 cells. Among all test samples, P1 and P4 were found to be the most potent and inhibited cell proliferation in a dose-dependent manner after 48 h of treatment (Fig. 2a & 2b). At 20 μg/ml, P4 inhibited the growth and proliferation to almost 99% (***P < 0.001) while P1 showed 56.57% (***P < 0.001) inhibition in MCF-7 cells. The IC_50_ values calculated were 14.25 ± 0.84 and 8.05 ± 0.22 μg/ml for P1 and P4, respectively. Both P1 and P4 were more potent than standard drug doxorubicin (IC_50_ value;16 ± 0.01 μg/ml) (Fig. 2c). As shown in Fig. 3, treatment with P1 and P4 led to apoptosis related morphological changes in the cell, such as cell shrinkage and roundness of the cell. Phase contrast microscopy images clearly show a decrease in cell count in the treatment groups as compared to control. These results suggest that proteins from NS seeds exhibited significant inhibitory activity against MCF-7 cell line.

### Analysis of pro-apoptotic and anti-apoptotic genes

**Fig. 4:**
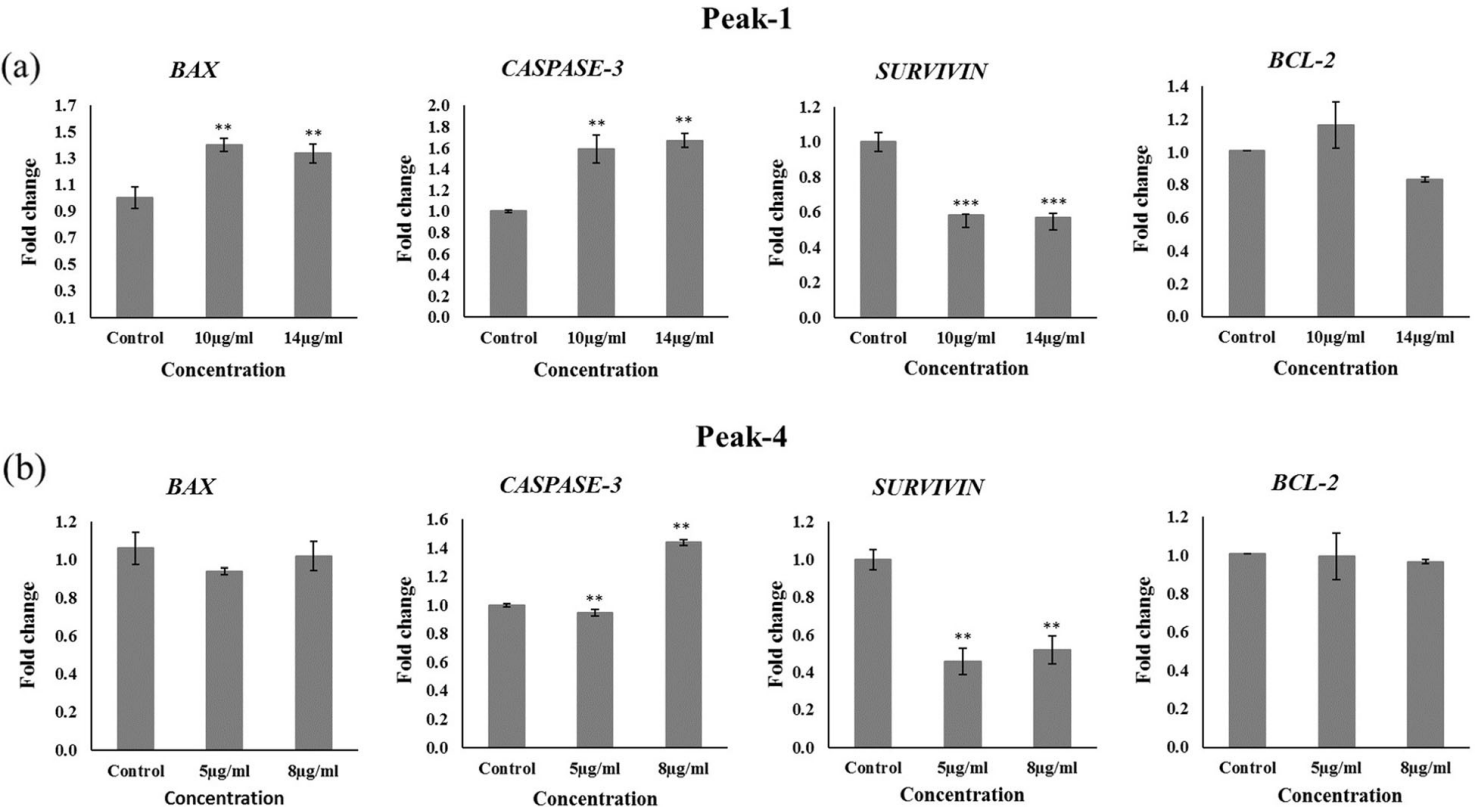
The effect of *Nigella sativa* seed proteins on the expression of pro-apoptotic and anti-apoptotic genes. Bar graphs show fold change in gene expression levels of *BAX*, *CASPASE-3*, *SURVIVIN,* and *BCL-2* after treatment with (**a**) P1 and (**b**) P4. The data were normalized with GAPDH and represented as a mean of triplicates for each dose ± S.D. The statistically significant values are labeled as *P < 0.05, ** < 0.01 and ***p < 0.001 as compared to the untreated control

To investigate whether P1 and P4 were responsible for inducing apoptosis in MCF- 7 cells, we examined the expression levels of pro-apoptotic (*BAX* and *CASPASE-3*) and anti-apoptotic (*SURVIVIN* and *BCL-2*) genes (Fig. 4). The intrinsic pathway is strictly regulated by bax to bcl-2 ratio. An increase in *BAX* expression triggers mitochondria to release cytochrome c that, in return, activates caspases, while bcl-2 inhibits the release [24]. Here, we observed a significant 1.4-fold (**P < 0.01) change upregulation in *BAX* and a 0.8 (**P < 0.01) fold change downregulation in *BCL-2* gene expression after treatment with P1 at dose 10 and 14 μg/ml, respectively (Fig. 4a). Subsequently, the expression of *CASPASE-3* increased significantly to 1.7-fold change (**P < 0.01) at 14 μg/ml in P1. The calculated *BAX* / *BCL-2* ratio, together with significant upregulation in the key executioner, *CASPASE-3* indicates the presence of intrinsic/extrinsic pathway of apoptosis compared to control cells. In contrast, treatment with P4 did not show any change in bax to bcl-2 ratio when compared to untreated control cells. However, *CASPASE-3* gene expression was found to be upregulated significantly by 1.4-fold change (**P < 0.01) at IC_50_ dose of 8 μg/ml, which may be due to activation of apoptosis by extrinsic pathway alone*. SURVIVIN*, a member of family inhibitors of apoptosis (IAPs), was also significantly downregulated by both P1 and P4.

### Identification of proteins in P1 and P2 by LC-MS/MS

To identify the proteins present in P1 and P4, in-solution digestion was performed, followed by LC-MS/MS, and a mass list obtained was searched against the UniProt database using the Mascot search engine. A list of proteins identified is presented in Tables 1 & 2. A total of 11 proteins were identified in P1 while 24 proteins in P4. Identified proteins from P1 and P4 represented a wide pI range from 5.5 to 11.4 and 4.7 to 9.9, respectively, whereas mass range from 11.4 to 111 kDa and 4.3 to 93.2 kDa, respectively. In P1, the highest significant score of 101 was observed for fructose-bisphosphate aldolase 8, while histone H4 had the highest 43.7% coverage. However, in P4, Protein SLE1 was the widely covered protein with 83.9% coverage, and Dehydrin Rab15 presented the highest significant score of 189.

**Table 1:** The list of proteins identified by Mascot in peak 1(P1)

Identified Protein	Accession No.	Score	Sequence Coverage %	Molecular Mass kDa	*pI*
Fructose-bisphosphate aldolase 8	Q9LF98	101	20.4	38.8	6.0
Glyceraldehyde-3-phosphate dehydrogenase 2	Q7FAH2	86	32.9	36.9	6.3
Glucose and ribitol dehydrogenase homolog	Q75KH3	81	21.3	32.4	5.7
Peroxygenase	Q9SQ57	81	5.30	27.6	5.5
Enolase 2	Q9LEI9	77	17.3	48.1	5.9
Histone H4	Q71V09	75	43.7	11.4	11.4
Ubiquitin-60S ribosomal protein	B9DHA6	61	34.4	14.9	9.9
NADPH-dependent aldehyde reductase 1	Q9FZ42	60	17.0	31.6	6.1
Proteasome subunit alpha type-7	O24030	59	22.0	28.6	8.4
Phosphoenolpyruvate carboxylase	Q02909	58	18.5	111.0	5.6
Proteasome subunit beta type 4	Q7DLR9	57	39.0	27.7	6.0

**Table 2:** The list of proteins identified by Mascot in peak 4 (P4)

Identified Protein	Accession No.	Score	Sequence Coverage %	Molecular Mass kDa	*pI*
Dehydrin Rab15	Q00742	189	21.5	15.7	9.9
Carrot ABA-induced in somatic embryos 3	Q5KTS7	180	32.7	12.1	6.1
Protein SLE1	I1N2Z5	150	83.9	12.2	5.3
Em-like protein GEA6	Q02973	85	14.1	9.9	6.7
Ubiquitin-NEDD8-like protein RUB1	Q9SHE7	146	28.8	17.4	5.7
Phosphoglycerate kinase	P12783	135	21.7	42.1	5.6
60S ribosomal protein L12	O50003	99	14.5	17.9	9.0
ATP synthase subunit beta	Q01859	91	25.2	59.0	5.9
Enolase 1	Q9LEJ0	88	34.6	48.0	5.5
Enolase	P42896	82	33.9	48.1	5.5
Bifunctional enolase 2/transcriptional activator	P25696	43	33.3	47.9	5.5
Elongation factor 1-alpha	P34824	84	12.1	49.4	9.2
Late embryogenesis abundant protein 31	Q9LJ97	78	40.8	26.7	4.7
Em protein	P04568	66	41.9	9.9	5.5
Embryonic abundant protein 1	P46520	66	26.3	10.1	5.5
Em protein H2	Q08000	58	44.1	9.9	5.5
Triosephosphate isomerase	P12863	72	27.3	27.2	5.5
Thionin NsW1	C0HJH9	69	28.6	4.3	9.6
ADP-ribosylation factor GTPase-activating protein	Q5W7F2	60	10.5	93.2	8.3
Late embryogenesis abundant protein 1	O49816	56	34.5	19.0	8.6
Alcohol dehydrogenase class-P	P06525	56	32.2	41.8	5.8
ATP synthase subunit alpha	P05493	55	23.3	55.2	6.0
Vicilin-like antimicrobial peptides 2–3	Q9SPL3	54	13.9	74.6	6.5
Glyceraldehyde-3-phosphate dehydrogenase	P34922	53	23.4	36.7	6.5

### Hydropathy profile

**Fig. 5:**
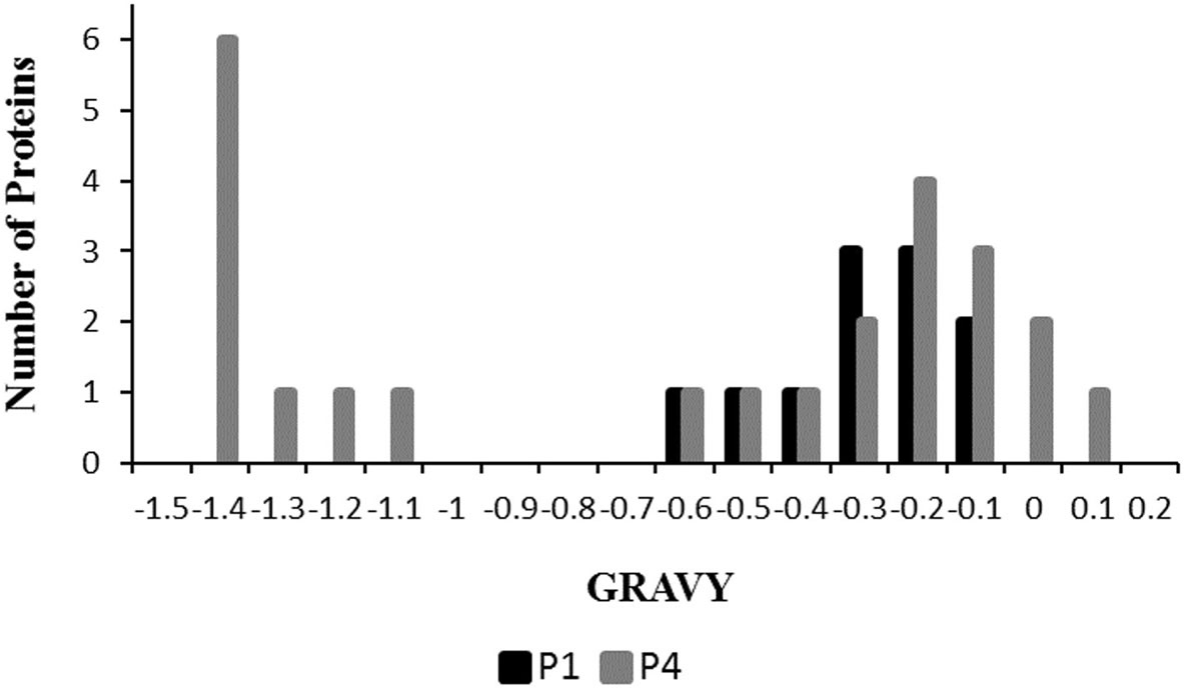
Distribution of the grand average of hydropathicity (GRAVY) value for all the identified proteins in P1 and P4. Positive and negative values represent hydrophobic and hydrophilic proteins, respectively

The grand average of hydropathicity (GRAVY) value for all the identified proteins from P1 and P4 were calculated, as shown in Fig. 5. The proteins with positive scores are hydrophobic, and negative scores are hydrophilic. In P4, both positive and negative values were observed, indicating the presence of both hydrophobic and hydrophilic proteins with hydrophilic being in the majority. While in P1, GRAVY scores between − 0.6 and − 0.1 indicate only hydrophilic proteins.

### Gene ontology annotation

**Fig. 6:**
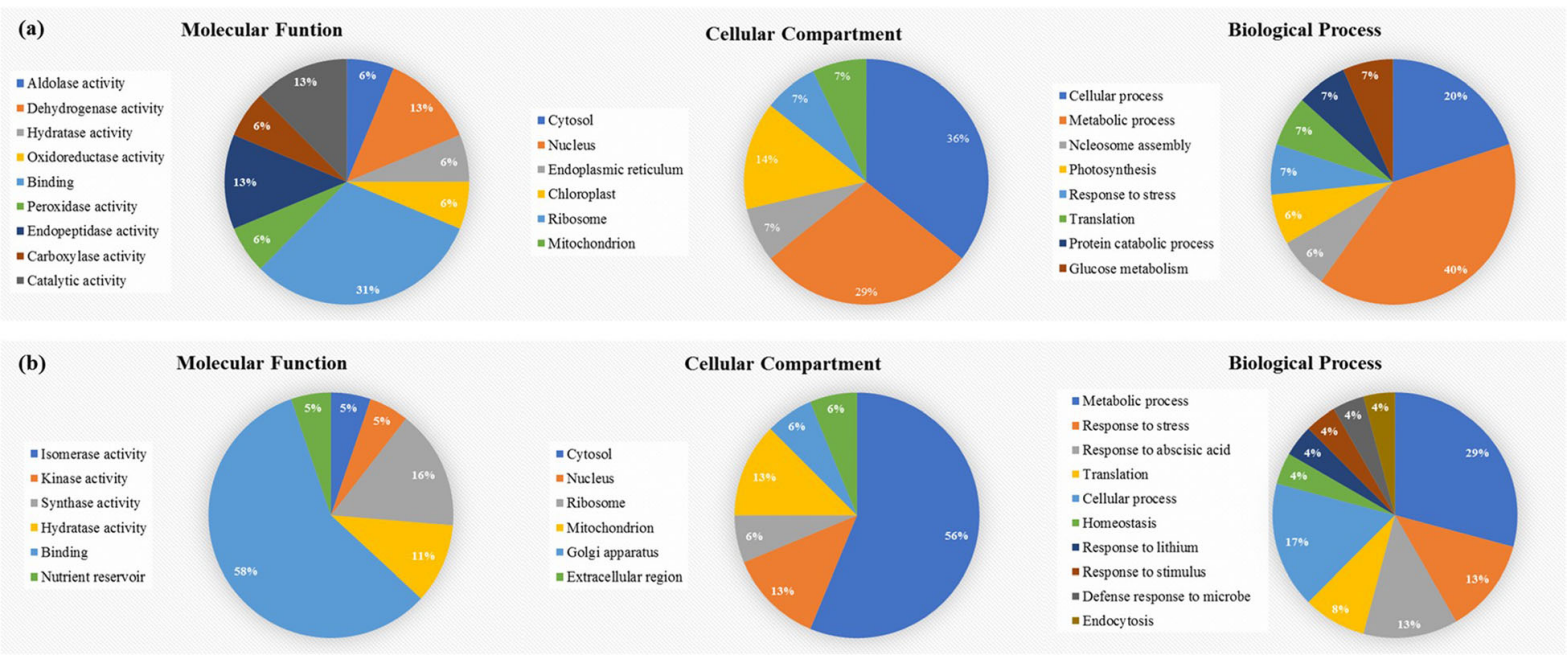
Gene ontology pie charts of all the identified proteins in (**a**) peak 1 and (**b**) peak 4. All proteins were classified based on their molecular function, cellular compartment, and biological process

The identified proteins were further categorized by UniProt GO annotation based on their cellular localization, biological process, and molecular functions, as shown in Fig. 6. Most of the proteins were found to be localized in cytosol (36%), nucleus (29%) and chloroplast (14%) in P1 (Fig. 6a) while cytosol (56%), nucleus (13%) and mitochondrion (13%) in P4 (Fig. 6b). In the category of biological process 40 and 29% of the identified proteins were involved in metabolic processes in P1 and P4, respectively. Other major proteins in P1 and P4 were associated with response to stress (20%), and cellular process (17%), respectively. Further molecular functional classification revealed for P1 was, binding activity (31%), endopeptidase activity (13%), dehydrogenase activity (13%), other catalytic activity (13%) and for P4 binding activity (58%), synthase activity (16%) and hydratase activity (11%).

## Discussion

Since ancient times natural products have been used for the treatment of various ailments and remains to be a widely used form of complementary and alternative medicine (CAM) alongside allopathic drugs [25, 26]. Recent technological advancement has brought protein and peptide-based drugs into the spotlight. Plants have played a historically proven role in drug development and continue to offer an inexhaustible supply of novel drug leads [27]. Although 60% of the approved small molecule based anticancer drugs are plant-derived yet in reality, only a handful of plants have been explored for the presence of antitumor proteins and peptides [5]. NS is one of the most extensively studied plants. Due to its wide spectrum of pharmacological activities, NS stands among the top-ranked herbal medicine [28]. Therefore, it is worthwhile to evaluate the anticancer potential of NS seed proteins.

In the present study, we successfully demonstrated the cytotoxic potential of partially purified NS seed proteins against MCF-7 cells. The crude seed proteins were successfully separated into seven peaks on the gel filtration chromatography column. The tris/glycine SDS PAGE analysis of both crude and FPLC fractions revealed the presence of multiple protein bands. Next, we screened all the peaks (P1, P2, P3, P4, P5, P6, and P7) and crude seed proteins for their cytotoxic effect. P1 and P4 found to be the most active against MCF-7, whereas no inhibitory activity was observed in the case of crude and peaks (P2, P3, P5, P6, and P7). Further, P1 and P4 were tested at different concentrations for IC_50_ calculations. Results showed dose-dependent inhibition in MCF-7 cells after 48 h of treatment. The IC_50_ value, a dose at which 50% inhibition observed were calculated as 14.25 ± 0.84 μg/ml for P1 and 8.05 ± 0.22 μg/ml for P4. These results were further supported by phase contrast microscopy images, which also showed a decrease in cell count with the increase in dose. Our results are in accord with the previous anticancer studies reported from proteins of medicinal plants *Gynura procumbens* (longevity spinach) [29]*, Corydalis cava* (turkey corn) [30], *Theobroma cacao* (cocoa) [31] and *Morinda pubescens* (mulberry) [32].

In the subsequent experiment, we investigated the possible cell killing mechanism exerted by P1 and P4 on MCF-7 cells. Apoptosis, programmed cell death, is an important cellular process that is responsible for eliminating the mutated cancer cells [33]. It can be induced by extrinsic or intrinsic pathways. The intrinsic mitochondrial pathway is under the regulation of pro-apoptotic (e.g., Bax) and anti-apoptotic (e.g., Bcl-2) genes. The major player in apoptosis are caspases that are responsible for the execution of apoptosis. Evasion from apoptosis is the most prominent hallmark of cancer cells [34]. Hence, activation of apoptosis is an intriguing approach to fight against malignancy. Herein, apoptosis was induced in human breast MCF-7 cancer cells by modulating the expression of *BAX*, *CASPASE-3, SURVIVIN,* and *BCL-2* genes (Fig. 4). At 14 μg/ml in P1, a significant upregulation in *BAX* and a downregulation in *BCL-2* led to an increase in the expression of *CASPASE- 3*, an executioner of apoptosis. *BAX* to *BCL-2* ratio is important in determining the fate of the cell for life or death. Treatment with P1 led to a dose-dependent increased in *BAX* to *BCL-2* ratio, indicating the presence of a potential anticancer agent. While in the case of P4, no change in expression of *BAX* and *BCL-2* genes were observed; however, the *CASPASE-3* gene expression was increased significantly, indicating the activation of the intrinsic pathway of apoptosis alone. Survivin, a member of inhibitors of apoptosis (IAPs), is a multifunctional protein that promotes angiogenesis, inhibits apoptosis, and controls cell proliferation [35]. Overexpression of survivin is associated with almost all types of breast cancer, including MCF-7 [36, 37]. We observed a significant decrease in *SURVIVIN* gene expression, which suggests apoptosis as a possible killing mechanism in MCF-7 following treatment with P1 and P4.

The active protein fractions were further analyzed using LC-MS/MS, and data acquired were searched against the Mascot database for protein identification. In P1, 11 proteins were identified, and the majority of them were located in the cytosol and hydrophilic in nature. Three proteins out of eleven, namely, proteasome subunit alpha type-7, proteasome subunit beta type-4, and ubiquitin-60S ribosomal protein, are from plant ubiquitin-proteasome system. Plant proteasome follows two routes for degradation of damaged or misfolded proteins, ubiquitin dependent proteolysis, and ubiquitin independent proteolysis [38]. The latter is crucial for the removal of oxidized proteins produced by reactive oxygen species [39, 40]. With its additional RNAase activity, these proteins offer protecting role against plant viruses such as the tobacco mosaic virus (TMV) [41, 42]. Nevertheless, the involvement of these proteins in the degradation of proteins and RNA of cancer cells needs further validation. Another identified protein, fructose-bisphosphate aldolase 8, belongs to the plant defense related proteins. This protein has been previously reported from *Gynura procumbens* [29] and *Corydalis cava* tuber [30] for their antiproliferative effect on MDA-MB-231 and HeLa cells, respectively. Other identified proteins in P1 were associated with plant metabolic and cellular processes, and their role as antitumor has not been studied yet.

In the case of P4, 24 proteins, including one peptide, were identified. The majority of the identified proteins were hydrophilic in nature, located in the cytosol, and involved in the metabolic process. Thionin NsW1, an antimicrobial peptide (AMP) from NS was also identified in P4. Thionins from different plants are well studied for their anticancer activity, such as thionin from *Pyrularia pubera* (buffalo nut) was reported as cytotoxic to B16 mouse melanoma and HeLa cervical cancer cell lines [43]. Thionin, namely viscotoxins from *Viscum spp* induced apoptosis by upregulating the expression of caspase 3 in human lymphocytes [44]. Furthermore, thionin, Thi2.1 from *Arabidopsis thaliana* (mouse-ear cress) inhibited the growth of MCF-7, HeLa, and A549 cancer cell lines [45]. Another identified plant defense peptide vicilin in P4, also known as antimicrobial peptide (AMP), has been reported active against a variety of microbial species [46]. Recently, Gupta et al., have demonstrated the cytotoxic activity of mungbean vicilin protein hydrolysate against MDA-MB-231 and MCF-7 breast cancer cell lines. They have also studied the inhibitory effect of vicilin peptides on the angiogenesis converting enzyme (ACE) [47]. Herein, the antiproliferative and apoptotic effect by P4 could be attributed by vicilin and/or thionin. Whereas, the pharmacological significance of the other identified proteins in P4 has not been evaluated.

Our study is the preliminary demonstration of the anticancer effects of NS seed proteins. The active protein fractions consist of a mixture of plant proteins. In the future, a study will be designed to evaluate the effect of individual isolated protein and in combination on cancer cells. The synergistic effect exhibited by the mixture of proteins in P1 and P4 is an important finding. In fact, many effective phytomedicines in markets are a crude mixture of proteins such as bromelain, which is a crude mixture of various enzymes, including endopeptidases, glucosidase, cellulase, peroxidase, escharase, and phosphatase, from *Ananas comosus* (pineapple) fruit and stem [48]. Bromelain is used to treat pain, burns, inflammation, osteoarthritis, cancer, thrombophlebitis, and sport related injuries [49–53].

## Conclusion

Presented studies successfully demonstrated the antiproliferative and apoptosis inducing potential of NS seed proteins on MCF-7 cells. The synergistic behavior by a mixture of protein in NS protein fractions is an interesting finding. This is the first study from NS seed in this context and may open an avenue in the future for plant-based protein pharmaceuticals. However, further studies are needed to isolate and purify the individual protein responsible for the In-vitro anticancer effect and to evaluate the underlying mechanism.

## Data Availability

Data and materials are available from authors on reasonable request.

## Abbreviations

ACE: Angiogenesis converting enzyme
AMP: Antimicrobial peptide
CAM: Complementary and alternative medicine
FDR: False discovery rate
FPLC: Fast protein liquid chromatography
GO: Gene ontology
GRAVY: Grand average of hydropathy
NS: *Nigella sativa*
P1: Peak-1
P4: Peak-4
PBS: Phosphate-buffered saline
TMV: Tobacco mosaic virus
UniProtKB: UniProt Knowledgebase
